# Postoperative Surgical Site Infection among Patients with Caesarean Delivery in the Department of Obstetrics and Gynaecology in a Tertiary Care Centre: A Descriptive Cross-sectional Study

**DOI:** 10.31729/jnma.8185

**Published:** 2023-06-30

**Authors:** Poonam Koirala, Ishita Koirala, Sunita Bajracharya, Hima Rijal, Asmita Ghimire, Anita Chamlagain

**Affiliations:** 1 Department of Obstetrics and Gynaecology, Tribhuvan University Teaching Hospital, Maharajgunj, Kathmandu, Nepal; 2 Nepal Medical College and Teaching Hospital, Jorpati, Kathmandu, Nepal

**Keywords:** *hypertension*, *prevalence*, *surgical site infection*

## Abstract

**Introduction:**

Surgical site infections are the commonest nosocomial infections following surgeries. They not only increase the morbidity and mortality following surgeries but also have a great impact both psychologically and financially. The aim of this study was to find out the prevalence of postoperative surgical site infection among patients with caesarean delivery in the Department of Obstetrics and Gynaecology in a tertiary care centre.

**Methods:**

This descriptive cross-sectional study was conducted in the Department of Obstetrics and Gynaecology of a tertiary care centre after taking ethical approval from the Institutional Review Committee (Reference number: 495(6-11)E2 077/078). Data from 1 July 2021 to 1 July 2022 were collected between 1 September 2022 to 30 November 2022 from the hospital records. All the pregnant women undergoing caesarean delivery during the study period were included. Convenience sampling method was used. Point estimate and 95% Confidence Interval were calculated.

**Results:**

Out of 1326 patients who underwent caesarean delivery, surgical site infection was seen in 38 (2.86%) (1.96-3.76, 95% Confidence Interval). Among 38 women, anaemia was seen in 11 (28.94%), diabetes mellitus in 6 (15.79%) and hypertension in 5 (13.16%).

**Conclusions:**

The prevalence of surgical site infection following caesarean delivery was found to be lower than other studies done in similar settings.

## INTRODUCTION

Postoperative surgical site infections (SSI) are an important health care associated infection (HAI) and are one of the most frequent causes of postoperative morbidity.^1^ World Health Organization shows that SSIs are most frequently reported type of HAI in low- and middle-income countries with a pooled incidence of 11. 8 episodes of SSI per 100 surgical procedures.^1^ Caesarean delivery (CD) is a major obstetrical surgical procedure aiming to save the lives of mothers and fetuses.^2^

The rate of SSI ranges from 3-15% worldwide.^3,4^ Although rate of SSI has decreased in past three decades due to better antibiotic prophylaxis , however due to rising incidence of caesarean delivery SSI rate has also been in increasing trend. Post-caesarean SSI may increase maternal morbidity and mortality. It prolongs maternal hospitalization and also lead to other socioeconomic implications.^5^

The aim of this study was to find out the prevalence of postoperative surgical site infection among patients with caesarean delivery in the Department of Obstetrics and Gynaecology in a tertiary care centre.

## METHODS

This descriptive cross-sectional study was conducted in the Department of Obstetrics and Gynaecology of Tribhuvan University Teaching Hospital, Maharajgunj, Kathmandu, Nepal after obtaining ethical clearance from the Institutional Review Committee (Reference number: 495(6-11)E2 077/078). Data from 1 July 2021 to 1 July 2022 were collected between 1 September 2022 to 30 November 2022 from the hospital records. Patients undergoing caesarean delivery were included whereas those with caesarean hysterectomy, exploratory laparotomy or women with surgery done outside our hospital and those who reported late after 30 days of infection were excluded from the study. Convenience sampling method was used. The sample size was calculated using the following formula:


$$\begin{array}{ll} \text{n} & ={\text{Z}}^{2}\times \frac{\text{p}\times \text{q}}{{\text{e}}^{\text{2}}}\\ & =1.96^{2}\times \frac{0.50\times 0.50}{0.03^{2}}\\ & =1068 \end{array}$$

Where,

n = minimum required sample size
Z = 1.96 at 95% Confidence Interval (CI)
p = prevalence taken as 50% for maximum sample size calculation
q = 1-p
e = margin of error, 3%

The minimum required sample size was 1068. However, the final sample size taken was 1326.

Cases were quantified as SSI based on criteria as per Centers for Disease Control and Prevention (CDC). Surveillance for SSI was based on the CDC definition. The CDC has defined SSI to standardize data collection for the National Nosocomial infections Surveillance (NNIS).^6^ SSIs are classified as incisional SSIs and organ/space SSIs, which effect the rest of the body other than the body wall layers.^7^

The study variables included demographic features, duration of rupture of membranes prior to CD, presence of diabetes mellitus (DM), hypertension, anaemia and heart disease. Anaemia was defined as preoperative hemoglobin <10 gm%, number of blood transfusion required during surgery or prior to surgery. The other factors like type of surgery emergency or elective, type of anaesthesia, operative time, administration of prophylactic antibiotics, were recorded. Prophylactic antibiotic 1 g ceftriaxone 15-30 min prior to caesarean delivery surgery or intraoperatively and followed by 2 g metronidazole after delivery as per standard hospital protocol was given in all patients.^6,7^ The wound swab culture sent in all patients who developed SSI was noted. All the data was collected from the admission book of female surgical ward and respected files of the patient.

The collected data were entered and analyzed using IBM SPSS Statistics version 22.0. Point estimate and 95% CI were calculated.

## RESULTS

Among 1326 patients who underwent CD, 38 (2.86%) (1.96-3.76, 95% CI) patients developed SSI. Majority of patients who developed SSI were of parity ≥2, 27 (71.05%) and term gestation, 25 (65.78%). Out of total, 12 (31.57%) had prelabour rupture of membranes and 19 (50%) had duration of rupture of membranes >10 hours prior to caesarean delivery (Table 1).

**Table 1:** Obstetric characteristics of patients with SSI following CD (n= 38).

Variable	n (%)
**Age (years)**	
19-24	2 (5.26)
25-30	21 (55.26)
31-36	11 (28.94)
37-42	4 (10.52)
**Parity**	
Primiparous	11 (28.94)
P2	16 (42.10)
P3	6 (15.78)
P≥3	5 (13.15)
Total	38 (100)
**Gestational age in weeks**	
<37	12 (31.57)
37-42	25 (65.78)
>42	1 (2.63)
Duration of rupture of membranes prior to caesarean section	
Intact	11 (28.94)
0-10 hrs	8 (21.05)
>10-20 hrs	11 (28.94)
≥21 hrs	8 (21.05)
**Comorbidities**	
Overt DM/gestational DM	6 (15.78)
Hypertension	5 (13.15)
Hypertension with DM	3 (7.89)
Heart disease	1 (2.63)
Thrombocytopenia	2 (5.26)
Anaemia	11 (28.94)
None	10 (26.31)

Emergency caesarean delivery was the most common one in 28 (73.68%) and elective in 10 (26.31%) (Table 2).

**Table 2:** Surgery related factors in patients with obstetric SSI (n= 38).

Variable	n (%)
**Type of surgery**	
Elective	10 (26.31)
Emergency	28 (73.68)
**Type of anaesthesia**	
Spinal	29 (76.31)
General anaesthesia	9 (23.68)
**Duration of surgery**	
<1 hour	14 (36.84)
1-2 hour	20 (52.63)
>2 hours	4 (10.52)
**Type of skin incision**	
Pfannensteil	27 (71.05)
Vertical	11 (28.94)
**Blood transfusion**	
Yes	11 (28.94)
No	27 (71.05)
**Duration of postoperative stay (days)**	
1-7	6 (15.78)
>7-14	28 (73.68)
>14	4 (10.52)

Fetal distress was the mostcommon reason forcaesarean delivery 11 (28.94%) in the patients with SSI(Table 3).

**Table 3:** Indication of caesarean delivery with SSI (n = 38)

Indications	n (%)
Previous caesarean delivery	5 (13.15)
Fetal distress	11 (28.94)
Prolonged labour	4 (10.52)
Hypertensive disorders	7 (18.42)
Uncontrolled diabetes with hypertension	4 (10.42)
Intrauterine growth retardation	1 (2.63)
Antepartum haemorrhage	2 (5.26)
Heart disease	4 (10.52)

Among patients with SSIs, 16 (42.10%) patients needed wound resuturing, 4 (10.52%) patients needed resuturing twice, whereas others 22 (57.89%) were managed conservatively. In culture, 16 (42.10%) samples were sterile, whereas 9 (23.68%) had *Staphylococcus aureus* positive (Table 4).

**Table 4:** Organisms found on culture (n= 38).

Organism	n (%)
*Staphylococcus aureus*	9 (23.68)
*Escherichia coli*	6 (15.78)
*Pseudomonas aeruginosa*	4 (10.52)
*Citrobacter freundii*	2 (5.26)
*Acinetobacter isolated*	1 (2.63)
*Sterile*	16 (42.10)

## DISCUSSION

Among 1326 patients who underwent CD, 38 (2.86%) patients developed SSI. Inspite, of an abundance of antibiotic prophylaxis and aseptic measures followed during surgery, SSI is still the commonest nosocomial infection following surgery. It not only increases financial burden but is the leading cause of morbidity and mortality among patients undergoing surgery.

SSIs rates can vary worldwide, with 9% in a study from India.^8^ Another study in Ujjain, Madhyapradesh, India showed SSI rate of 7.84%,^9^ however our rates are lower compared to that as they included both obstetric and gynecological surgeries, whereas a study from USA^10^ showed 5.5% SSI rates. Variety of studies in Nepal show different SSI rates studies such as 27.7% in Nepal Nobel hospital and 2.76% at Patan hospital.^11,12^ On the other hand in low resource settings in Tanzanian the SSI rates are 48%.^13^

The low prevalence in the present study could be as we have taken here only the admitted cases, whereas many cases have been missed as they were managed on the outpatient service conservatively. This variation may also depend on the sample size, proper use of standard antibiotic prophylaxis, comorbidities present, referred cases to the tertiary care centre and aseptic measures used for surgical procedures and various risk factors associated with SSI. SSI has always been associated with risk factors and in a study from USA showed that various risk factors have been found to predict post caesarean SSI.^14^ A study from Eastern Nepal shows mean age of 24.04 years in post caesarean SSI.^15^

In the present study majority of women were of parity >2, term gestation and had prolonged duration of rupture of membranes prior to caesarean delivery. Moreover, the majority of patients had rupture of membranes more than 10 hrs prior to CD. This can be explained by the loss of cervical mucus plug and barrier effect of fetal membranes and amniotic fluid which helped in preventing the ascending infection. This is quite similar to a study done in Tanzania which showed prolonged rupture of membranes more than 8 hours or longer is a significant risk factor for post caesarean wound infection.^16^

Majority of the SSI in present study occurred in emergency CD and most of the patients had duration of surgery more than 1 hr. Various studies such as study from Pakistan also found emergency caesarean section as risk factor for development of SSI.^17^ This can be explained as emergency surgeries are mostly unplanned and are associated with other risk factors like anaemia, multiple vaginal examinations, antibiotic prophylaxis less than 30 min prior to surgery, referred cases with comorbidities, late arrival of patients to seek medical care and inadequate blood transfusion prior to surgery. Although antibiotic prophylaxis was given in all the patients either prior or during the surgery, but in emergency cases it is delayed and given during the surgery which also might be inadequate for the patients resulting in SSI in emergency surgeries.

Emergency caesarean deliveries are usually done without proper preoperative work up. Moreover patients with poor health conditions are usually associated with other comorbidities which further increase the prevalence of SSIs.

In the present study duration of surgery more than 1 hr, hypertension and diabetes were most commonly found in patients with SSI following CD. Diabetes has been found to be associated with SSI following surgery and poorly controlled diabetes impairs host immune response and delays the re-epithelialization of wounds.^18^ Due to less collagen production in diabetics, the wound healing is generally impaired and slow, thus leading to SSIs in such patients. Similarly, patients with hypertensive disorder of pregnancy also have been shown to have 2.9 times the risk for SSI.^19^

Preoperative anaemia is also considered as an important risk factor for predicting SSI and has been shown by several studies. A study from India showed that anaemia is the commonest risk factor for SSI.^20^ In the present study, majority of patients needed blood transfusion either prior or during the CD. The healing capacity of tissues is reduced in anaemic patients as well as there is hypo-oxygenation in the tissues. Thus these patients are more prone for infection. Moreover allogenic blood transfusion induces immunosuppression which further predisposes to postoperative infection.

In our study coagulase negative *Staphylococcus aureus* was the commonest pathogen found followed by *Escherichia coli* and *Citrobacter.* Overall 14 (36.8%) patients needed wound re-suturing which was done under aseptic measures whereas other were managed conservatively.

This study has certain limitations. It is a single centered study with a small sample size. Also all the patients with SSI could not be included as many patients with SSI were managed on outpatient basis due to shortage of availability of beds. Moreover, being a tertiary care centre patients come from all over Nepal for surgeries so actual data of SSI may be lacking as patients return home after discharge with very few only reporting to hospital.

## CONCLUSIONS

The prevalence of surgical site infection following caesarean delivery was lower in comparison to studies done in similar settings. The identification of co-morbidities is essential to improve maternal and fetal outcomes and minimise maternal morbidity and mortality.
